# R534C mutation in hERG causes a trafficking defect in iPSC-derived cardiomyocytes from patients with type 2 long QT syndrome

**DOI:** 10.1038/s41598-019-55837-w

**Published:** 2019-12-16

**Authors:** Fernanda C. P. Mesquita, Paulo C. Arantes, Tais H. Kasai-Brunswick, Dayana S. Araujo, Fernanda Gubert, Gustavo Monnerat, Danúbia Silva dos Santos, Gabriel Neiman, Isabela C. Leitão, Raiana A. Q. Barbosa, Jorge L. Coutinho, Isadora M. Vaz, Marcus N. dos Santos, Tamara Borgonovo, Fernando E. S. Cruz, Santiago Miriuka, Emiliano H. Medei, Antonio C. Campos de Carvalho, Adriana B. Carvalho

**Affiliations:** 1 Carlos Chagas Filho Institute of Biophysics, Federal University of Rio de Janeiro. Avenida Carlos Chagas Filho 373, Bloco G, Rio de Janeiro, RJ 21941-902 Brazil; 2 National Center for Structural Biology and Bioimaging, Federal University of Rio de Janeiro. Avenida Carlos Chagas Filho 373, Bloco M, Rio de Janeiro, RJ 21941-902 Brazil; 3 Institute of Biomedical Sciences, Federal University of Rio de Janeiro. Avenida Carlos Chagas Filho 373, Bloco F, Rio de Janeiro, RJ 21941-902 Brazil; 4 FLENI Foundation, Sede Escobar. Ruta 9, Km 53, Belen de Escobar, BA B1625 Argentina; 5 National Institute of Cardiology, Rua das Laranjeiras 374, Rio de Janeiro, RJ 22240-006 Brazil; 6 Pontifical Catholic University of Parana. Rua Imaculada Conceição 1155, Curitiba, PR 80215-901 Brazil; 7 National Institute for Science and Technology in Regenerative Medicine. Avenida Carlos Chagas Filho 373, Bloco M, Rio de Janeiro, RJ 21941-902 Brazil

**Keywords:** Ion transport, Mechanisms of disease, Induced pluripotent stem cells, Stem-cell differentiation

## Abstract

Patient-specific cardiomyocytes obtained from induced pluripotent stem cells (CM-iPSC) offer unprecedented mechanistic insights in the study of inherited cardiac diseases. The objective of this work was to study a type 2 long QT syndrome (LQTS2)-associated mutation (c.1600C > T in KCNH2, p.R534C in hERG) in CM-iPSC. Peripheral blood mononuclear cells were isolated from two patients with the R534C mutation and iPSCs were generated. In addition, the same mutation was inserted in a control iPSC line by genome editing using CRISPR/Cas9. Cells expressed pluripotency markers and showed spontaneous differentiation into the three embryonic germ layers. Electrophysiology demonstrated that action potential duration (APD) of LQTS2 CM-iPSC was significantly longer than that of the control line, as well as the triangulation of the action potentials (AP), implying a longer duration of phase 3. Treatment with the I_Kr_ inhibitor E4031 only caused APD prolongation in the control line. Patch clamp showed a reduction of I_Kr_ on LQTS2 CM-iPSC compared to control, but channel activation was not significantly affected. Immunofluorescence for hERG demonstrated perinuclear staining in LQTS2 CM-iPSC. In conclusion, CM-iPSC recapitulated the LQTS2 phenotype and our findings suggest that the R534C mutation in KCNH2 leads to a channel trafficking defect to the plasma membrane.

## Introduction

Since induced pluripotent stem cells (iPSC) were first described^1,2^, they have been widely used for disease modeling^3,4^ and are a powerful tool for understanding disease mechanisms^5–8^. Importantly, iPSC-derived cardiomyocytes have reproduced several monogenic cardiac disorders that may result in sudden cardiac death (SCD)^9,10^.

The leading cause of SCD in the young-adult population (less than 35 years old) are arrhythmic syndromes with or without structural heart alterations^11^. Long QT syndrome (LQTS) is the most common inherited disorder in autosomal dominant cardiac diseases^12,13^.

LQTS is clinically characterized by a prolongation of the QT interval (corrected QT interval greater than 470 ms in women and 450 ms in men) in conjunction with syncope, ventricular fibrillation or SCD^14^. The prevalence of congenital LQTS is 1 in 2,500^15,16^ and it has been associated with variants in at least 16 genes^17^. In general, a single mutation is present in one of the associated genes and the most common genotypes are type 1 LQTS (mutations in KCNQ1), type 2 (in KCNH2), type 3 (in SCN5A) and type 5 (in KCNE1). Together, they represent more than 90% of the genetically confirmed cases and each type has distinct triggers, penetrance and responsiveness to therapy^11,13^.

KCNH2 codes for the pore-forming subunit of cardiac potassium channel hERG, responsible for the rapid component of the delayed rectifier repolarizing current (I_Kr_) in the heart. Loss of function mutations in this channel increase action potential duration (APD) as well as the QT interval^18,19^.

LQTS2 has been modeled *in vitro* using *Xenopus* oocytes or HEK293 cells to dissect the underlying genetic causes of hERG dysfunction^20–23^. However, these exogenous expression systems do not recapitulate the complex interactions between the various types of ion channels present in a human cardiomyocyte. Current gene editing technologies make it possible to correct or introduce mutations in iPSC, controlling for patient genetic background and epigenetic variability^24^.

In this study, we have generated iPSC from two LQTS2 patients with c.1600C > T, p.R534C mutation and introduced this same mutation in a control iPSC line. These cell lines were differentiated into cardiomyocytes and characterized by electrophysiology.

## Results

### Generation of induced pluripotent stem cells and genome editing

Peripheral blood mononuclear cells (PBMNC) were isolated from a healthy male donor (24 years old, CTRL-iPSC) and 2 donors with a diagnosis of familial LQTS2 with a heterozygous R534C mutation (female, 44 years old, LQTS2-iPSC1; and male, 17 years old, LQTS2-iPSC2). PBMNC were enriched for erythroblasts and, after 12 days, cells were reprogrammed (Supplementary Fig. S1a). The first colonies with pluripotent characteristics emerged ~15 days post-transduction. iPSCs were selected based on morphology (rounded colonies, well-defined colony edges, and high nucleus-to-cytoplasm ratio) (Supplementary Fig. S1b), expanded and characterized (Supplementary Fig. S1c-e and S2). These clones had a normal karyotype (Supplementary Fig. S1c) and, to confirm the presence of the mutation after reprogramming, exon 7 of KCNH2 was genotyped. We observed a normal sequence in our CTRL-iPSC and detected the point mutation (c.1600C > T) in heterozygosis (Supplementary Fig. S1d) in LQTS2-iPSC1 and LQTS2-iPSC2.

To investigate the effect of the R534C KCNH2 mutation in an identical genetic background, a homologous recombination strategy was used in our CTRL-iPSC to insert this mutation. Using the CRISPR/Cas9 system, we designed a single guide RNA (sgRNA) to precede a 5′-NGG PAM region to cleave the target (Supplementary Fig. S3a) and cloned the sgRNA in a plasmid that contained CRISPR/Cas9 (Supplementary Fig. S3b). The repair template used was a single-stranded DNA oligonucleotide (ssODN) containing the KCNH2 single nucleotide mutation (Supplementary Fig. S3c). The plasmid and the ssODN were nucleofected into the CTRL-iPSC and puromycin-resistant colonies were isolated manually (Supplementary Fig. S1b). Homologous recombination in homozygosis was confirmed by DNA sequencing of one clone (Supplementary Fig. S1d). The clone maintained its normal karyotype (46 XY) (Supplementary Fig. S1c) after homologous recombination.

Cells expressed pluripotency markers (Supplementary Fig. S1e and S2a) and differentiated spontaneously into the three embryonic germ layers (Supplementary Fig. S2b). We observed characteristic nuclear staining for OCT4, SOX2 and NANOG and cytoplasmic staining for LIN28, TRA1-60 and TRA1-81 in all of our iPSC lines (Supplementary Fig. S2a). Spontaneous differentiation resulted in the expression of Nestin (ectoderm), Brachyury (mesoderm) and alpha-fetoprotein (AFP, endoderm), providing additional evidence of pluripotency (Supplementary Fig. S2b).

### LQTS2 cardiomyocytes exhibit prolonged action potential duration

**Figure 1:**
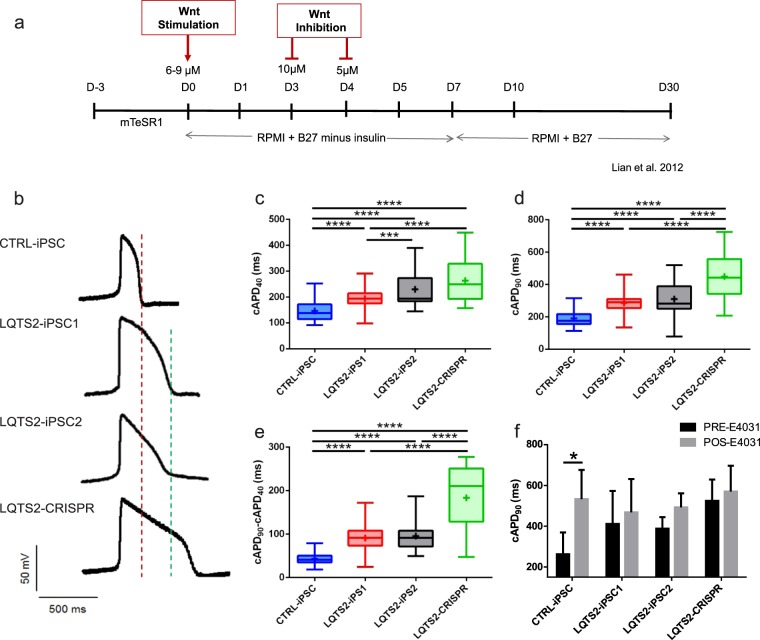
Differentiation and electrophysiology of iPSC-derived cardiomyocytes. (**a**) Schematic diagram demonstrating the main steps of the differentiation procedure. (**b**) Representative action potential recordings of spontaneously contracting ventricular-like cardiomyocytes. Note the red line that marks the end of phase 3 for CTRL-iPSC and the green line that marks the end of phase 3 for LQTS2-iPSC1 and LQTS2-iPSC2. (**c**,**d**) Our analysis demonstrates that action potential duration of LQTS2-iPSC1, 2 and CRISPR was significantly longer than that of CTRL-iPSC, as was the triangulation of action potentials (**e**), implying a longer duration of phase 3. CTRL-iPSC (n = 116); LQTS2-iPSC1 (n = 59); LQTS2-iPSC2 (n = 77); LQTS2-CRISPR (n = 20) from 8 independent differentiations for each cell line. Box plots represent 1st quartile, median and 3rd quartile. Whiskers represent minimum and maximum values. “+” represents the mean for each cell line. (**f**) Treatment with E4031 caused APD prolongation only in CTRL-iPSC, indicating that hERG does not contribute to repolarization in LQTS2- and CRISPR-iPSC (n = 4). Bars represent mean and SEM. *p < 0.05, ****p < 0.0001.

After confirming that iPSC lines were pluripotent, they were submitted to cardiac differentiation (Fig. 1a). On day 7, we observed the first beating areas. Cells were cultured for 30 days before electrophysiology experiments.

To evaluate the impact of the mutation on action potential duration (APD), action potentials (AP) were recorded in ventricular-like cells and classified according to the criteria defined by Ma *et al*.^25^. Representative APs of cardiomyocytes derived from CTRL-iPSC, LQTS2-iPSC1, LQTS2-iPSC2, and LQTS2-CRISPR are shown in Fig. 1b. We observed a significant prolongation of APD in cardiomyocytes derived from LQTS2 iPSC lines compared to CTRL-iPSC. Corrected APD at 40% of repolarization (cAPD40) (Fig. 1c) and cAPD90 (Fig. 1d) were significantly longer in cardiomyocytes from LQTS2-iPSC1 (193.5 ± 4.78 and 285.4 ± 7.09 ms), LQTS2-iPSC2 (229.8 ± 8.03 and 309.7 ± 10.57 ms) and LQTS2-CRISPR (263.0 ± 18.53 and 448.4 ± 30.45 ms) when compared to CTRL-iPSC (145.9 ± 3.74 and 189.4 ± 4.37 ms). cAPD90 of LQTS2-CRISPR cardiomyocytes was significantly longer than LQTS2-iPSC1 (Fig. 1d). Triangulation (cAPD90 – cAPD40) was higher in all LQTS2 cardiomyocytes when compared to control, implying a more prolonged phase 3 of the AP (Fig. 1e). In addition, triangulation of LQTS2-CRISPR cardiomyocytes was significantly longer compared to all other cell lines (CTRL-iPSC: 42.95 ± 1.12, LQTS2-iPSC1: 90.82 ± 3.15, LQTS2-iPSC2: 95.72 ± 3.70 and LQTS2-CRISPR: 183.5 ± 17.86 ms) (Fig. 1e). Treatment with the I_Kr_ inhibitor E4031 in a separate subset of cells caused a significant prolongation of cAPD90 only in CTRL-iPSC cardiomyocytes (before E4031: 262.45 ± 53.64, after E4031: 532.58 ± 71.70 ms, n = 4) (Fig. 1f), indicating that hERG does not contribute to repolarization in LQTS2- and CRISPR-iPSC cardiomyocytes. Maximum diastolic potential (MDP) was similar between all cell lines (CTRL-iPSC: −45.39 ± 2.74, LQTS2-iPSC1: −45.56 ± 2.70, LQTS2-iPSC2: −48.55 ± 1.95 and LQTS2-CRISPR: −40.85 ± 5.83 mV).

### Channel activation is not affected by the R534C mutation

**Figure 2:**
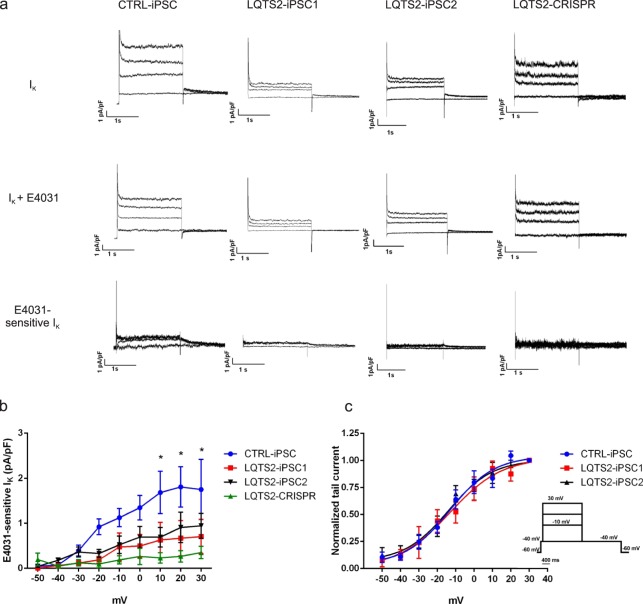
Patch clamp of iPSC-derived cardiomyocytes. (**a**) Representative patch clamp recordings of potassium currents before and after treatment with E4031 in all groups. These currents were subtracted to obtain I_Kr_ (E4031-sensitive I_K_). (**b**) I-V graph showing I_Kr_ current densities for all cell lines. Control cardiomyocytes had significantly higher currents at 10, 20 and 30 mV compared to LQTS2 cardiomyocytes (*p < 0.05). (**c**) Average I_Kr_ tail current normalized to the maximal current following repolarization to −40 mV in CTRL-iPSC, LQTS2-iPSC1 and LQTS2-iPSC2. Voltage protocol used for patch clamp recordings of the representative currents is also shown.

To investigate the biophysical properties of the hERG channel in LQTS2 cardiomyocytes, we measured I_Kr_ density and tail currents by patch clamp before and after treatment with E4031. We observed that CTRL-iPSC displayed an E4031-sensitive current (1.02 ± 0.23 pA/pF, n = 6) and that these currents were significantly smaller in LQTS2-iPSC1 (0.37 ± 0.09 pA/pF, n = 8), LQTS2-iPSC2 (0.52 ± 0.10 pA/pF, n = 10) and LQTS2-CRISPR (0.19 ± 0.03 pA/pF, n = 7) cardiomyocytes (Fig. 2a,b). Importantly, although I_Kr_ densities were reduced in all LQTS2 lines, the normalized tail currents were similar to those obtained from the control (Fig. 2c). V_1/2_ of the activation curves were also not different between experimental groups (CTRL-iPSC: −13.17 ± 3.41, LQTS2-iPSC1: −13.13 ± 5.63, LQTS2-iPSC2: −13.99 ± 4.19). This indicates that channel activation is not affected by the mutation.

### R534C mutation affects channel trafficking to the membrane

Since electrophysiology data strongly suggest that the mutation in KCNH2 does not affect channel gating, we conducted immunofluorescence for hERG to investigate if the mutation causes a channel trafficking defect to the plasma membrane.

**Figure 3:**
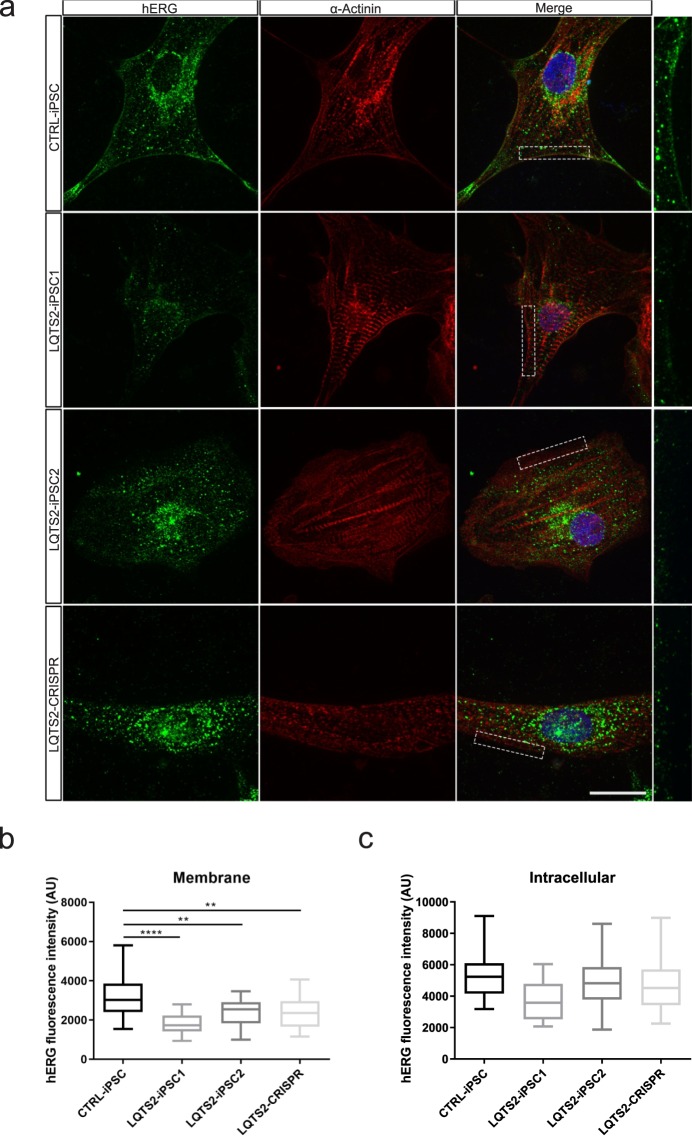
Characterization of hERG trafficking defect in LQTS2-iPSC cardiomyocytes. (**a**) Immunostaining for sarcomeric α-actinin (red) and hERG (green) in hiPSC-CM. Control cardiomyocytes exhibit membrane and intracellular hERG localization. A higher magnification of the membrane region marked by the dashed rectangle is shown on the right. Observe that, in LQTS2-iPSCs and LQTS2-CRISPR, hERG staining is reduced when compared to control. Quantification of hERG fluorescence intensity signal in the membrane (**b**) and intracellular space (**c**). CTRL-iPSC (n = 25); LQTS2-iPSC1 (n = 22); LQTS2-iPSC2 (n = 20); LQTS2-CRISPR (n = 17). Box plots represent 1st quartile, median and 3rd quartile. Whiskers represent minimum and maximum values. **p < 0.01, ****p < 0.0001. Scale bar: 10 µm.

hERG staining was prominently observed at the plasma membrane in cardiomyocytes from CTRL-iPSC, with diffuse staining all over the cell (Fig. 3a). In contrast, the levels of hERG in the membrane were reduced in LQTS2-iPSC1, LQTS2-iPSC2 and LQTS2-CRISPR (Fig. 3a,b). An evident intracellular accumulation with similar intensity was observed in the cardiomyocytes from LQTS2-iPSC1, LQTS2-iPSC2 and LQTS2-CRISPR (Fig. 3a). Analyzing hERG cellular localization, we observed a significant difference between CTRL-iPSC cardiomyocytes and LQTS2-derived cardiomyocytes at the membrane level (Fig. 3b), while no differences were present between experimental groups in the intracellular localization (Fig. 3c). These data suggest that the mutation indeed causes an hERG trafficking defect.

## Discussion

The present study demonstrated that iPSC-derived cardiomyocytes generated from patients recapitulated the LQTS2 phenotype. Moreover, if the R534C mutation is present in homozygosis, the phenotype is more severe. Our findings suggest that this mutation leads to a channel trafficking defect to the plasma membrane.

Although this mutation has already been described^26,27^, its mechanism of action remains unclear. Itoh and colleagues described a missense mutation in the S4 region of hERG in a Japanese LQTS2 family. Using a heterologous expression system in *Xenopus* oocytes, the authors suggested that the mutation caused a channel gating defect, shifting the voltage-dependence of hERG activation in a negative direction, reducing steady-state inactivation. Using a computer simulation, the authors also suggested that the mutation did not affect AP duration^22^. This finding is inconsistent with the LQTS2 phenotype, as pointed out in the editorial accompanying the original publication^28^. Roden & Balser predicted that, although the channel might be successfully expressed in the membrane of *Xenopus* oocytes, the major effect in mammalian cells might be a decrease in the number of channels in the membrane. Using the CM-iPSC model, our data demonstrated an average increase in cAPD90 of 50.7% and 63.5% in LQTS2 CM-iPSC1 and 2 respectively, and of 2.37-fold in CRISPR-modified CM-iPSC. By patch clamp, we observed significantly lower I_Kr_ density in LQTS2 and CRISPR-modified CM-iPSC with no shift in the activation curve. Therefore, iPSC-derived cardiomyocytes are more reliable than *Xenopus* oocytes for *in vitro* disease modeling of LQTS, although the latter can be excellent models to study isolated protein function.

Mutations in KCNH2 generally have a dominant negative effect, resulting in inappropriate protein maturation that causes a reduction of I_Kr_^29^. Trafficking-defects create abnormalities in the transport of ion channels from the endoplasmic reticulum (ER) to the membrane, leading to their degradation^30^. It has been demonstrated that the transient expression of the R534C KCNH2 mutation in HEK293 cells inhibits Golgi processing. Consequently, the protein remains in its non-glycosylated form, suggesting that this mutation leads to a trafficking-deficient phenotype^30^. Using immunofluorescence, we observed a reduced plasma membrane staining in our LQTS2 cardiomyocytes when compared to control cells. This intracellular localization is characteristic of an hERG trafficking defect according to another study using an iPSC model in which CM-iPSC harbored the A561V, A561P and IVS9-28A/G hERG mutations^31,32^.

CM-iPSC models are valuable tools to study ion channel variants that may be pathogenic and to identify the mechanisms by which mutations cause loss of function in the channel. These mutations can modify the number of channels expressed in the plasma membrane, the gating or the conductance of the channel^33^. CM-iPSC models have been widely used to define the characteristics of each mutation^29,31,34^. However, due to variability in iPSC line generation and overlap of electrophysiologic characteristics between control and mutated lines, it is increasingly important to introduce the variant in a control cell line to confidently attribute a pathogenic role to a specific mutation, as done in our experiments.

In conclusion, we have generated iPSC from two patients with LQTS2 and introduced this same mutation in a control iPSC. CM-iPSC recapitulated the LQTS2 phenotype and introduction of the mutation in homozygosis in our control iPSC line aggravated the phenotype. Our findings indicate that the R534C mutation in KCNH2 leads to a trafficking defect of the protein to the plasma membrane, which results in reduced I_Kr_, prolongation of AP duration and the prolonged QT interval observed in our patients.

## Methods

### Generation of patient-specific iPSC and differentiation into cardiomyocytes

Two patients with a heterozygous mutation in KCNH2 (c.1600C > T, p.R534C)^26^ were invited to donate 5 mL of peripheral blood after obtaining written informed consent. This study was approved by the National Institute of Cardiology’s ethics review board under number 27044614.3.0000.5272. All experiments were performed in accordance with relevant guidelines and regulations. Reprogramming of erythroblasts was conducted with Sendai Virus as previously reported^35^. Briefly, mononuclear cells were cultivated in enrichment medium for erythroblasts and, after 12 days, cells were infected with CytoTune-iPS 2.0 Sendai Reprogramming Kit (Thermo Scientific). iPSCs were maintained in DMEM/F12, GlutaMAX supplement, with 20% KSR, 1% penicillin-streptomycin (P/S), 100 µM NEEA, 0.1 mM β-mercaptoethanol, 10 ng/mL of bFGF.

iPSCs were differentiated into cardiomyocytes according to previously published protocols^36,37^ (Fig. 1a). Briefly, 3 × 10^5^ iPSCs were plated with 1% Matrigel hESC-Qualified Matrix (Corning) and cultured in mTeSR1 (STEMCELL Technologies) for 3 days. On day 0, cells were transferred to RPMI 1640 (Gibco) with B-27 Supplement minus insulin (Gibco) and 6–9 μM of CHIR99021 (R&D Systems). After 24 hours, CHIR99021 was removed and, on days 3 and 4, Wnt signaling was inhibited with 10 and 5 μM of XAV939 (R&D Systems), respectively. On day 7, we observed beating areas and cells were transferred to RPMI 1640 with B-27 Supplement until day 30.

### Karyotype analysis

iPSC were evaluated using an adapted protocol previously reported^38^. Briefly, 10 mg/mL of KaryoMax Colcemid solution (Gibco) was added to cells and incubated at 37 °C for 1 hour to block cell division in mitotic metaphase. Cells were detached and treated with 6 mL of 0.075 M KCl with HEPES, followed by incubation at 37 °C for 20 minutes and fixation with methanol:acetic acid (3:1) solution. G-bands were obtained by heating the slides at 60 °C and treating with a trypsin solution (0.002 g/mL) for 5 seconds. The staining procedure was carried out using Giemsa (1:20) solution, producing trypsin and Giemsa (GTG) bands. Twenty metaphases were analyzed using a Leica microscope (DM2000) and the LUCIA (Laboratory Imaging) software. Karyotype is described according to ISCN^39^.

### EB formation and spontaneous differentiation

For embryoid body (EBs) formation, iPSCs were incubated with 1 mg/mL of collagenase I (Sigma-Aldrich) in PBS with Ca^++^ and Mg^++^ and 20% FBS for 20 min at 37 °C. Subsequently, cells were dissociated to form small aggregates with 0.05% Trypsin-EDTA (Gibco) and mechanical scrapping. Aggregates were plated in ultra-low attachment plates (Corning) with the following basal culture medium: StemPro®-34 SFM (Gibco), 1% glutamine, 1% P/S, 150 μg/mL of transferrin (Roche Life Sciences), 0.039 μL/mL of monothioglycerol (Sigma-Aldrich), 50 μg/mL ascorbic acid (Sigma-Aldrich) and 1 μM of Y-27632 dihydrochloride (R&D Systems). For spontaneous differentiation into the three embryonic germ layers, EBs were cultured in suspension for 7 days in basal culture medium. Then, EBs were transferred back to adherent plates and cultured for another 7 days for immunofluorescence^40^.

### Gene edition

To insert a point mutation in our control iPSC we used the plasmid pSpCas9(BB)-2A-Puro V2.0 (Addgene®, Cat. #62988) to generate our 20-nt guide sequence within the single guide RNA (sgRNA). The single-stranded DNA oligonucleotide (ssODN) template was designed 50 bp away from the double-strand break (DSB) site^41^ (Supplementary Table 1). Ten μM of the ssODN template and 500 ng of the Cas9 plasmid were nucleofected in the control iPSC line using the Neon Transfection System (Invitrogen). Puromycin-resistant (0.5 μg/mL) colonies were picked and sequenced to confirm the introduction of the mutation by homologous recombination.

### Immunofluorescence

Cells were plated in glass slides pre-treated with Matrigel hESC-Qualified Matrix (Corning) and cultured in their respective media. After 3 days, samples were fixed in 4% paraformaldehyde for 20 min at room temperature. The following antibodies were used after three 5 min permeabilizations with 0.3% Triton X-100 in PBS: OCT4, SOX2 and NANOG (Cell Signaling). The following antibodies were used after one 5 min permeabilization with 0.01% Triton X-100 in PBS: TRA-1–60, LIN28, Brachyury, AFP (Cell Signaling), and Nestin (Millipore). Primary antibodies were incubated at 4 °C for 12 h. Then, cells were washed and incubated for 2 h at room temperature with Cy3-AffiniPure Donkey Anti-Rabbit (Jackson Immunoresearch) and/or AlexaFluor 488 Goat Anti-Mouse (Thermo Scientific) secondary antibodies associated with TO-PRO-3 (Thermo Scientific) for nuclei counterstaining. For cardiac proteins, we used a 3-day protocol. On day 1, we used the anti-KCNH2 (hERG) antibody (1:200, Abcam), 5 min permeabilization with 0.01% Triton X-100 in PBS and samples were incubated at 4 °C for 12 h. On day 2, cells were washed and incubated for 2 h at room temperature with Alexa Fluor® 488 Goat Anti-Rabbit secondary antibody (Thermo Scientific). Samples were re-fixed in 4% paraformaldehyde for 20 min at room temperature. Anti-α-Actinin antibody (Sigma-Aldrich) was used after three 5 min permeabilizations with 0.3% Triton X-100 in PBS and slides were incubated at 4 °C for 12 h. Then, cells were washed in PBS and incubated for 2 h at room temperature with Cy3-AffiniPure Goat Anti-Mouse secondary antibody (Jackson Immunoresearch) associated with TO-PRO-3 (Thermo Scientific) for nuclei counterstaining. Slides were mounted in ProLong Gold Antifade Mountant (Thermo Scientific) and visualized using the confocal microscope Zeiss LSM 510 Meta and Yokogawa Spinning Disk. Image processing was conducted with ZEN 2012 software. hERG fluorescent signal was quantified using ImageJ.

### RT-PCR

RNA was extracted using RNeasy Mini kit (QIAGEN) or miRNeasy Mini kit (Qiagen) and quantified by spectrophotometry with NanoDrop (Thermo Scientific). Reverse transcription was conducted using the High-Capacity Reverse Transcription Kit (Thermo Scientific) according to the manufacturer’s instructions. For PCR amplification, GoTaq® Flexi DNA Polymerase (Promega) and the primers described on Supplemental Table 1 were used. PCR products were analyzed by electrophoresis in agarose gels stained with SYBR® Safe (Thermo Scientific).

### DNA sequencing

Genomic DNA was extracted from the iPSCs and exons corresponding to the coding region of KCNH2 were amplified as previously published^26^. To amplify exon 7 of KCNH2, the PCRx Enhancer Reagent (Thermo Scientific) was added to the reaction, according to the manufacturer’s instructions. PCR products were purified by ExoSAP-IT PCR Product Cleanup Reagent (Affymetrix) and sequenced using sense or antisense primers, according to manufacturer’s instructions (primer sequences are available in Supplementary Table 1) in 3500-xL Genetic Analyzer with BigDye terminator v3.1 Cycle Sequencing Kit (Thermo Scientific).

### Electrophysiology

Recordings were obtained from cardiomyocytes after 30–45 days of differentiation.

Action potential recording method was adapted from a previously described protocol^42^. Spontaneously beating cardiomyocyte preparations were superfused with Tyrode’s solution containing (in mM) 140 NaCl, 5 KCl, 1.8 CaCl_2_, 1.0 MgCl_2_, 11 D-glucose and 5 HEPES (pH 7.4 adjusted with NaOH) at 37 ± 1 °C saturated with oxygen at a perfusion flow rate of 0.5 mL/min (Miniplus 3, Gilson). Transmembrane potential was recorded using glass microelectrodes (40–80 MΩ DC resistance) filled with 3 M KCl connected to a Microelectrode Amplifier (MultiClamp 700B, Molecular Devices). Amplified signals were digitized (1440 digidata A/D interface, Molecular Devices) and the following parameters were analyzed using LabChart 7.3 software (ADInstruments): action potential duration at 40% repolarization (cAPD40), cAPD90 (both corrected by beat rate), and triangulation (cAPD90 - cAPD40) from at least 10 consecutive action potentials for each cell. Measures were obtained automatically. The starting point of the action potential was determined by the software as a 10% variation in voltage from maximum diastolic potential.

I_Kr_ was recorded by whole-cell patch clamp at 36° ± 1 °C using borosilicate glass capillaries (3–5 MΩ DC resistance) filled with a pipette solution containing (mM) 140 KCl, 1 MgCl_2_, 10 HEPES, 4 MgATP and 5 EGTA (pH 7.2 adjusted with KOH) connected to a patch-clamp Amplifier Axopatch 200B (Axon Instruments, Foster City, CA) with a perfusion flow rate of 2.0 mL/min (Miniplus 3, Gilson, France). The data signal was amplified and filtered at 1 kHz and digitized at 10 kHz (1440 digidata A/D interface, Molecular Devices); data acquisition and analysis were conducted with pClamp 10.2 software (Molecular Devices). The bath solution was Tyrode’s solution (identical to the one used for AP recordings except for CaCl_2_, which was reduced to 1.0 mM) containing 5 µM of Nicardipine and 30 µM of Chromanol 293B to block the I_Ca,L_ and I_Ks_, respectively. I_Kr_ was measured as a 10 μM E4031-sensitive current, by subtraction of the current recorded before and after E-4031 application. To measure I_Kr_ tail currents, the voltage protocol consisted of a holding potential of −60 mV, a pre-pulse to −40 mV (to block I_Na_ and I_Ca,T_) and 9 depolarizing 10 mV pulses clamping the membrane potential at −50 to +30 mV and back to −40 mV. The density of the currents was calculated by dividing current amplitude (pA) by cell membrane capacitance (pF).

### Statistical analysis

Data were analyzed using GraphPad Prism version 6. Differences between variables were considered significant when p value was less than 0.05. Baseline APD and fluorescence intensity were analyzed using One-way ANOVA with Bonferroni multiple comparisons test and are represented as boxplots with whiskers showing minimum and maximum values. APD data before and after E4031 were analyzed using paired t tests and are shown as mean ± SEM. Activation curves and V_1/2_ were obtained from normalized tail currents using Boltzmann’s sigmoidal function and are represented as mean ± SEM.

## Supplementary information

Supplementary Material

Full length PCR gels

## Data Availability

The datasets generated during and/or analyzed during the current study are available from the corresponding author on reasonable request.

## References

[1] Takahashi K (2007). Induction of pluripotent stem cells from adult human fibroblasts by defined factors. Cell.

[2] Yu J (2007). Induced pluripotent stem cell lines derived from human somatic cells. Science.

[3] Holmqvist S (2016). Creation of a library of induced pluripotent stem cells from Parkinsonian patients. NPJ Parkinsons Dis.

[4] Csöbönyeiová M, Danišovič Ľ, Polák Š (2016). Recent advances in iPSC technologies involving cardiovascular and neurodegenerative disease modeling. Gen. Physiol. Biophys..

[5] Omole AE, Fakoya AOJ (2018). Ten years of progress and promise of induced pluripotent stem cells: historical origins, characteristics, mechanisms, limitations, and potential applications. PeerJ.

[6] Kawser Hossain M (2016). Recent Advances in Disease Modeling and Drug Discovery for Diabetes Mellitus Using Induced Pluripotent Stem Cells. Int. J. Mol. Sci..

[7] Inoue H, Nagata N, Kurokawa H, Yamanaka S (2014). iPS cells: a game changer for future medicine. EMBO J..

[8] Liu Y, Deng W (2016). Reverse engineering human neurodegenerative disease using pluripotent stem cell technology. Brain Res..

[9] Yang C (2015). Concise Review: Cardiac Disease Modeling Using Induced Pluripotent Stem Cells. Stem Cells.

[10] van Mil A (2018). Modelling inherited cardiac disease using human induced pluripotent stem cell-derived cardiomyocytes: progress, pitfalls, and potential. Cardiovasc. Res..

[11] Fernández-Falgueras, A., Sarquella-Brugada, G., Brugada, J., Brugada, R. & Campuzano, O. Cardiac Channelopathies and Sudden Death: Recent Clinical and Genetic Advances. *Biology* **6** (2017). 10.3390/biology6010007 PMC5372000 28146053

[12] Nakano Y, Shimizu W (2016). Genetics of long-QT syndrome. J. Hum. Genet..

[13] Bezzina CR, Lahrouchi N, Priori SG (2015). Genetics of sudden cardiac death. Circ. Res..

[14] Waddell-Smith KE, Skinner JR (2016). Update on the Diagnosis and Management of Familial Long QT Syndrome. Heart, Lung and Circulation.

[15] Schwartz PJ (2009). Prevalence of the congenital long-QT syndrome. Circulation.

[16] Barsheshet A, Dotsenko O, Goldenberg I (2014). Congenital long QT syndromes: prevalence, pathophysiology and management. Paediatr. Drugs.

[17] Schwartz PJ, Ackerman MJ, Wilde AAM (2017). Channelopathies as Causes of Sudden Cardiac Death. Cardiac Electrophysiology. Clinics.

[18] Sanguinetti MC, Jiang C, Curran ME, Keating MT (1995). A mechanistic link between an inherited and an acquired cardiac arrhythmia: HERG encodes the IKr potassium channel. Cell.

[19] Trudeau MC, Leung LM, Roti ER, Robertson GA (2011). hERG1a N-terminal eag domain–containing polypeptides regulate homomeric hERG1b and heteromeric hERG1a/hERG1b channels: A possible mechanism for long QT syndrome. The Journal of General Physiology.

[20] Furutani M (1999). Novel mechanism associated with an inherited cardiac arrhythmia: defective protein trafficking by the mutant HERG (G601S) potassium channel. Circulation.

[21] Zhao JT (2009). Not all hERG pore domain mutations have a severe phenotype: G584S has an inactivation gating defect with mild phenotype compared to G572S, which has a dominant negative trafficking defect and a severe phenotype. J. Cardiovasc. Electrophysiol..

[22] Nakajima T (1999). Voltage-shift of the current activation in HERG S4 mutation (R534C) in LQT2. Cardiovasc. Res..

[23] McBride CM (2013). Mechanistic basis for type 2 long QT syndrome caused by KCNH2 mutations that disrupt conserved arginine residues in the voltage sensor. J. Membr. Biol..

[24] Bellin M (2013). Isogenic human pluripotent stem cell pairs reveal the role of a KCNH2 mutation in long-QT syndrome. The EMBO Journal.

[25] Ma J (2011). High purity human-induced pluripotent stem cell-derived cardiomyocytes: electrophysiological properties of action potentials and ionic currents. Am. J. Physiol. Heart Circ. Physiol..

[26] Ernesto C (2011). Investigation of ion channel gene variants in patients with long QT syndrome. Arq. Bras. Cardiol..

[27] Itoh T (1998). Genomic organization and mutational analysis of HERG, a gene responsible for familial long QT syndrome. Human Genetics.

[28] Roden DM, Balser JR (1999). A plethora of mechanisms in the HERG-related long QT syndrome Genetics meets electrophysiology. Cardiovasc. Res..

[29] Matsa E (2011). Drug evaluation in cardiomyocytes derived from human induced pluripotent stem cells carrying a long QT syndrome type 2 mutation. Eur. Heart J..

[30] Anderson CL (2006). Most LQT2 mutations reduce Kv11.1 (hERG) current by a class 2 (trafficking-deficient) mechanism. Circulation.

[31] Jouni, M. *et al*. Toward Personalized Medicine: Using Cardiomyocytes Differentiated From Urine‐Derived Pluripotent Stem Cells to Recapitulate Electrophysiological Characteristics of Type 2 Long QT Syndrome. *Journal of the American Heart Association* **4** (2015). 10.1161/JAHA.115.002159 PMC4599503 26330336

[32] Mehta A (2018). Identification of a targeted and testable antiarrhythmic therapy for long-QT syndrome type 2 using a patient-specific cellular model. European Heart Journal.

[33] Smith JL (2016). Molecular pathogenesis of long QT syndrome type 2. Journal of Arrhythmia.

[34] Sala L (2016). A new hERG allosteric modulator rescues genetic and drug-induced long-QT syndrome phenotypes in cardiomyocytes from isogenic pairs of patient induced pluripotent stem cells. EMBO Mol. Med..

[35] Yang, W. iPSC reprogramming from human peripheral blood using Sendai Virus mediated gene transfer. *StemBook*, 10.3824/stembook.1.73.1 (2014). 23785736

[36] Lian X (2013). Directed cardiomyocyte differentiation from human pluripotent stem cells by modulating Wnt/β-catenin signaling under fully defined conditions. Nat. Protoc..

[37] Lian X (2012). Robust cardiomyocyte differentiation from human pluripotent stem cells via temporal modulation of canonical Wnt signaling. Proc. Natl. Acad. Sci. USA.

[38] Borgonovo T, Vaz IM, Senegaglia AC, Rebelatto CLK, Brofman PRS (2014). Genetic evaluation of mesenchymal stem cells by G-banded karyotyping in a Cell Technology Center. Revista Brasileira de Hematologia e Hemoterapia.

[39] McGowan-Jordan, J., Simons, A. & Schmid, M. Iscn 2016: An International System for Human Cytogenomic Nomenclature 2016. (S. Karger AG, 2016).

[40] Mesquita FCP (2015). Generation of human iPS cell line ihFib3.2 from dermal fibroblasts. Stem Cell Res..

[41] Ran FA (2013). Genome engineering using the CRISPR-Cas9 system. Nature Protocols.

[42] Monnerat-Cahli G (2014). Toll-like receptor 4 activation promotes cardiac arrhythmias by decreasing the transient outward potassium current (Ito) through an IRF3-dependent and MyD88-independent pathway. J. Mol. Cell. Cardiol..

