# Supplementary material for: R534C mutation in hERG causes a trafficking defect in iPSC-derived cardiomyocytes from patients with type 2 long QT syndrome
Source: Sci Rep. 2019 Dec 16;9:19203. doi: 10.1038/s41598-019-55837-w (PMC6915575; doi:10.1038/s41598-019-55837-w)

Supplementary Material

R534C mutation in hERG causes a trafficking defect in iPSC-derived cardiomyocytes from patients with type 2 long QT syndrome

Fernanda C. P. Mesquita[1], Paulo C. Arantes[1], Tais H. Kasai-Brunswick[1,2], Dayana S. Araujo[1], Fernanda Gubert[1,3], Gustavo Monnerat[1], Danúbia S. dos Santos[1], Gabriel Neiman[4], Isabela C. Leitão[1], Raiana A. Q. Barbosa[1], Jorge L. Coutinho[5], Isadora M. Vaz[6], Marcus N. dos Santos[1], Tamara Borgonovo[6], Fernando E. S. Cruz[5], Santiago Miriuka[4], Emiliano H. Medei[1,2], Antonio C. Campos de Carvalho[1,2,5,7,*], Adriana B. Carvalho[1,2,7,*].

Supplementary Legends

Supplementary Fig. S1. Generation of LQTS2-iPSC. (a) Schematic overview of the protocol. Patient-specific iPSC were generated using peripheral blood mononuclear cells (PBMNC). Cells were cultivated in enrichment medium for erythroblasts and transduced with Sendai virus (SeV). The first colonies emerged 20-30 days post-infection. iPSCs from the two patients and a control were selected and expanded. (b) CTRL-iPSC, LQTS2-iPSC1, LQTS2-iPSC2 and LQTS2-CRISPR clones after 5 passages. (c) All iPSCs had a normal karyotype after 10 passages (CTRL-iPSC – 46,XY,inv(9)(p12q13)[20]; LQTS2-iPSC1 – 46,XX[20]; LQTS2-iPSC2 – 46,XY[20]; LQTS2-CRISPR – 46,XY,inv(9)(p12q13)[20]). (d) We did not detect the mutation (c.1600C>T) in control cells though it persisted after reprogramming in the cells from the two patients. In LQTS2-CRISPR, the mutation was successfully introduced in homozygosis. (e) All cell lines expressed pluripotency transcripts (OCT4, SOX2, NANOG, REX1, KLF4, DNMT3B, TDGF, TERT, GDF3, LIN28, NODAL) by RT-

PCR. GAPDH was used as endogenous control. Images were cropped from different gels. Full-length gels are included as supplementary material.

Supplementary Fig. S2. Characterization of iPSC by immunofluorescence. (a) All cell lines demonstrated characteristic staining of pluripotent markers by immunofluorescence (nuclear for OCT4, NANOG and SOX2 and cytoplasmic for LIN28, TRA-1-81 and TRA-1-60). (b) iPSCs showed spontaneous differentiation into the three embryonic germ layers (Nestin – ectoderm, Brachyury – mesoderm and alpha-fetoprotein – endoderm). The nuclei are stained in blue. Scale bars: 20 µm or 50 µm.

Supplementary Fig. S3. Gene edition strategy applied to insert the KCNH2 mutation in a normal iPSC. (a-c) Construction of the CRISPR/Cas9 system. The mutation detected in exon 7 of LQTS2 patients was selected (a) and the guide RNA (gRNA) was designed and cloned in the CRISPR/Cas9 plasmid (b) for gene edition. The single-stranded DNA oligonucleotide (ssODN) (c) was designed to insert the mutation (c.1600C>T) in the control line.

Supplementary Table 1. List of primers used to amplify pluripotency markers by RT-PCR, DNA sequencing and gene edition.

Primers	Forward<br>Reverse
**PLURIPOTENCY**	
OCT4	5’ - AGCCTGAGGGCGAAGCAGGA - 3’<br>5’ - CCCCAGGGTGAGCCCCACAT - 3’
SOX2	5’ - AGCTACAGCATGATGCAGGA - 3’<br>5’ - GGTCATGGAGTTGTACTGCA - 3’
NANOG	5’ - CAGCCCTGATTCTTCCACCAGTCCC - 3’<br>5’ - TGGAAGGTTCCCAGTCGGGTTCACC - 3’
REX1	5’ - CAGATCCTAAACAGCTCGCAGAAT - 3’<br>5’ - GCGTACGCAAATTAAAGTCCAGA - 3’
KLF4	5’ - TCTCAAGGCACACCTGCGAA - 3’<br>5’ - TAGTGCCTGGTCAGTTCATC - 3’
DNMT3B	5’ - TGCTGCTCACAGGGCCCGATACTTC - 3’<br>5’ - TCCTTTCGAGCTCAGTGCACCACAAAAC - 3’

TDGF	5’ - GATATCTCAGCAAACAAGTTTGCCA - 3’<br>5’ - GGCAGGTCACTCAGGTTATTGTTGC - 3’
GDF3	5’ - CTTATGCTACGTAAAGGAGCTGGG - 3’<br>5’ - GTGCCAACCCAGGTCCCGGAAGTT - 3’
LIN28	5’ - CAAAAGGAAAGAGCATGCAGAA - 3’<br>5’ - ATGATCTAGACCTCCAGAGTTGTAGC - 3’
NODAL	5’ - GGGCAAGAGGCACCGTCGACATCA - 3’<br>5’ - GGGACTCGGTGGGGCTGGTAACGTTTC - 3’
GAPDH	5’ - ACCATGGGGAAGGTGAAGGT - 3′<br>5’ - CATGGGTGGAATCATATTGG - 3’
**DNA SEQUENCING**	
KCNH2	5’ - CTCCTCCCTTGCCCCCCTTG - 3′<br>5’ - GATGCAGGCTAGCCAGTGCG - 3′
**GENE EDITION**	
sgRNA_KCNH2	5’ - CACCGTCGGGCTGCTGAAGACTGCG - 3’<br>5’ - AAACCGCAGTCTTCAGCAGCCCGAC - 3’
ssODN_M	CCCCCAGCTGATCGGGCTGCTGAAGACTGCGCGGCTGCTG<br>CGGCTGGTGTGCGTGGCGCGGAAGCTGGATCGCTACTCAG<br>AGTACGGCGCGGCCGTGCTG

Supplementary Fig. S1

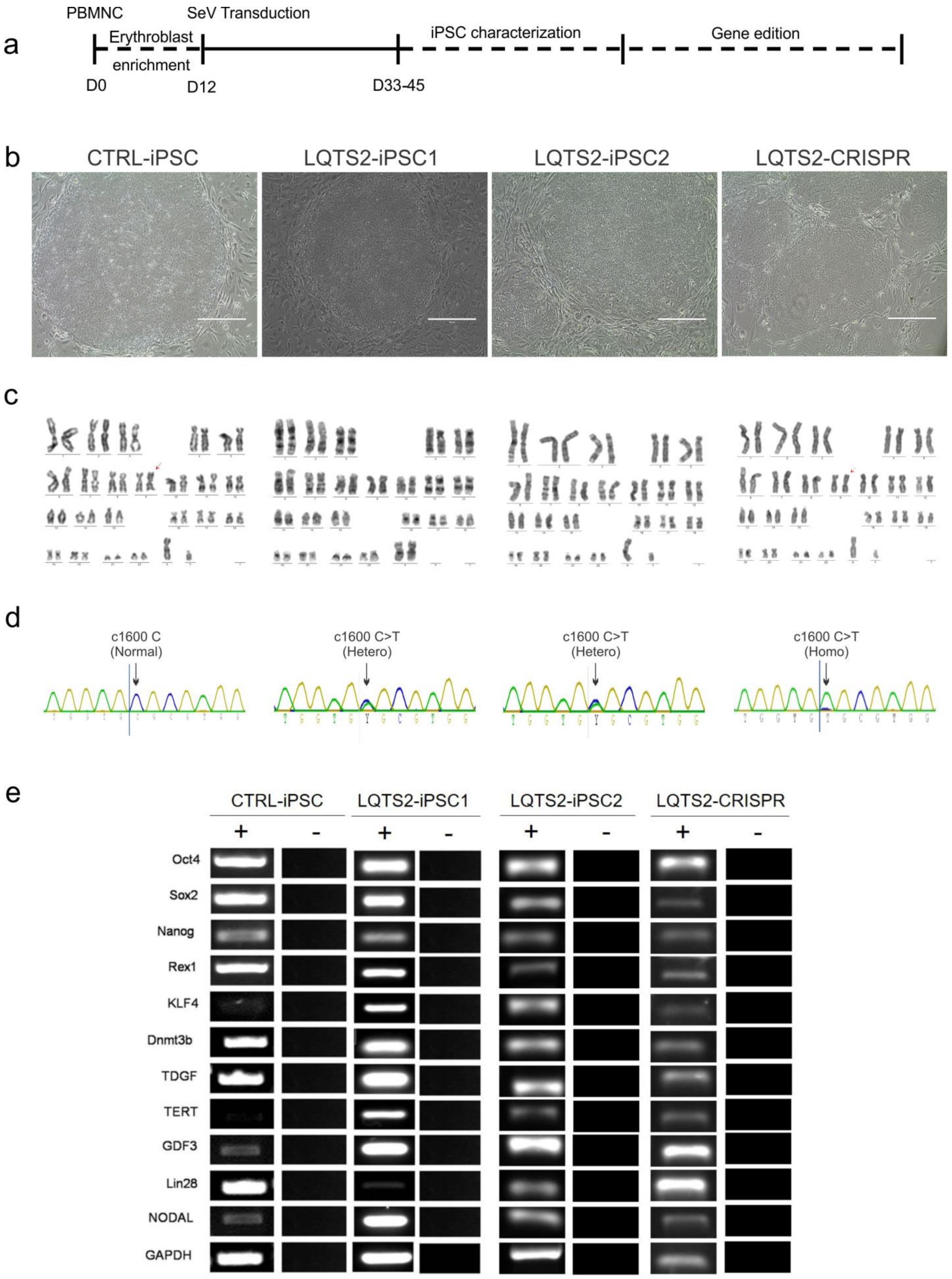

Supplementary Fig. S2

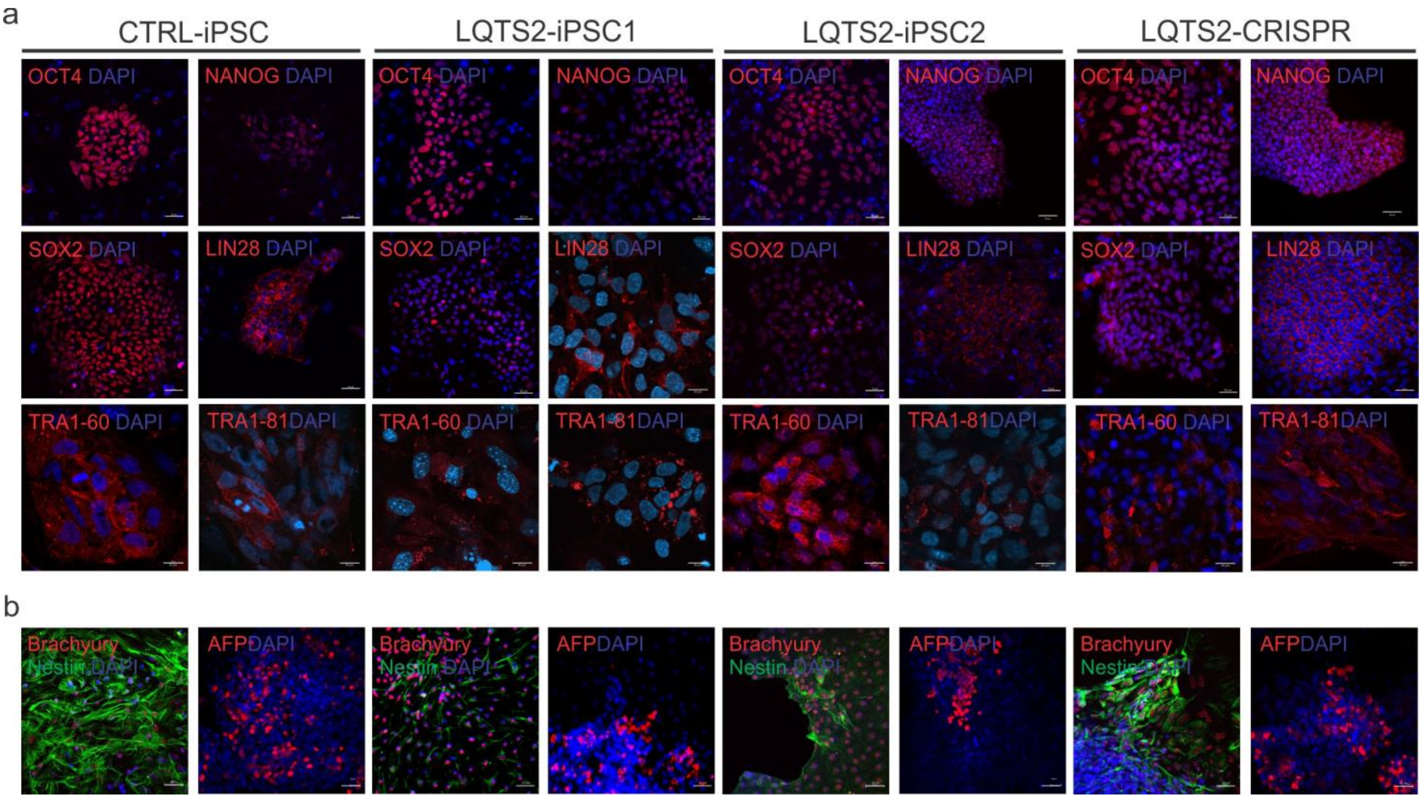

Supplementary Fig. S3

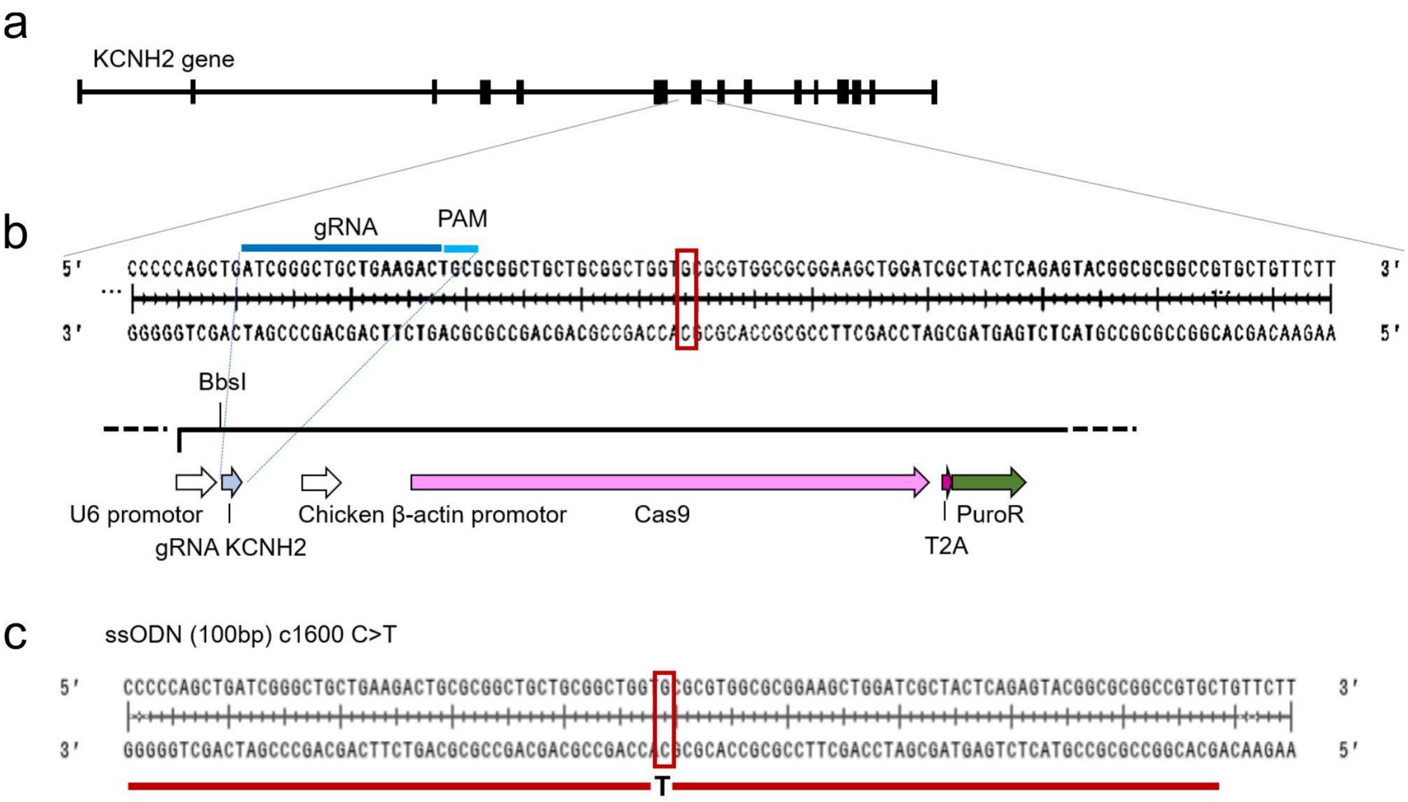

Supplement: Supplementary file 1 — Supplementary Material [file 41598_2019_55837_MOESM1_ESM.pdf]
