# Supplementary material for: R534C mutation in hERG causes a trafficking defect in iPSC-derived cardiomyocytes from patients with type 2 long QT syndrome
Source: Sci Rep. 2019 Dec 16;9:19203. doi: 10.1038/s41598-019-55837-w (PMC6915575; doi:10.1038/s41598-019-55837-w)

R534C mutation in hERG causes a trafficking defect in iPSC-derived cardiomyocytes from patients with type 2 long QT syndrome

Fernanda C. P. Mesquita[1], Paulo C. Arantes[1], Tais H. Kasai-Brunswick[1,2], Dayana S. Araujo[1], Fernanda Gubert[1,3], Gustavo Monnerat[1], Danúbia S. dos Santos[1], Gabriel Neiman[4], Isabela C. Leitão[1], Raiana A. Q. Barbosa[1], Jorge L. Coutinho[5], Isadora M. Vaz[6], Marcus N. dos Santos[1], Tamara Borgonovo[6], Fernando E. S. Cruz[5], Santiago Miriuka[4], Emiliano H. Medei[1,2], Antonio C. Campos de Carvalho[1,2,5,7,*], Adriana B. Carvalho[1,2,7,*].

CTRL-iPSC

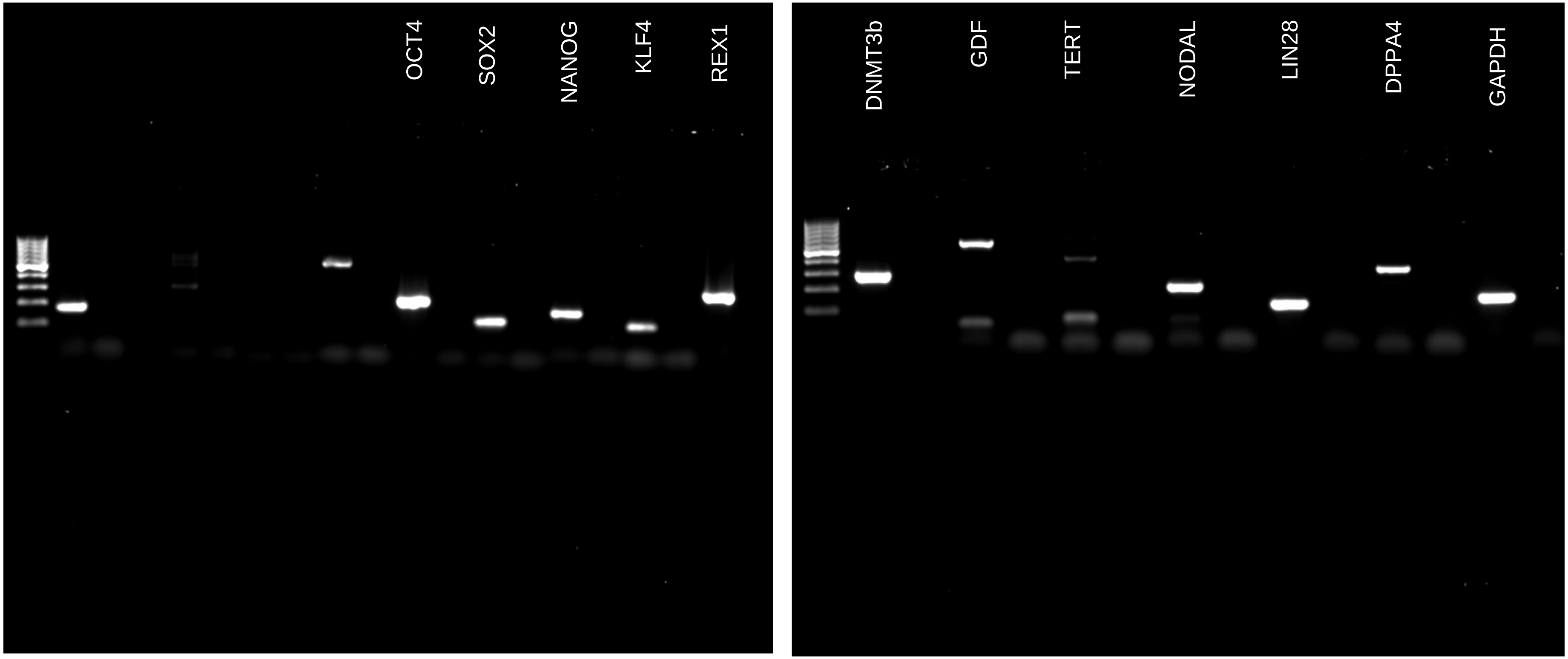

OCT4
SOX2
NANOG
KLF4
REX1
DNMT3b
GDF
TERT
NODAL
LIN28
DPPA4
GAPDH

LQTS2-iPSC1

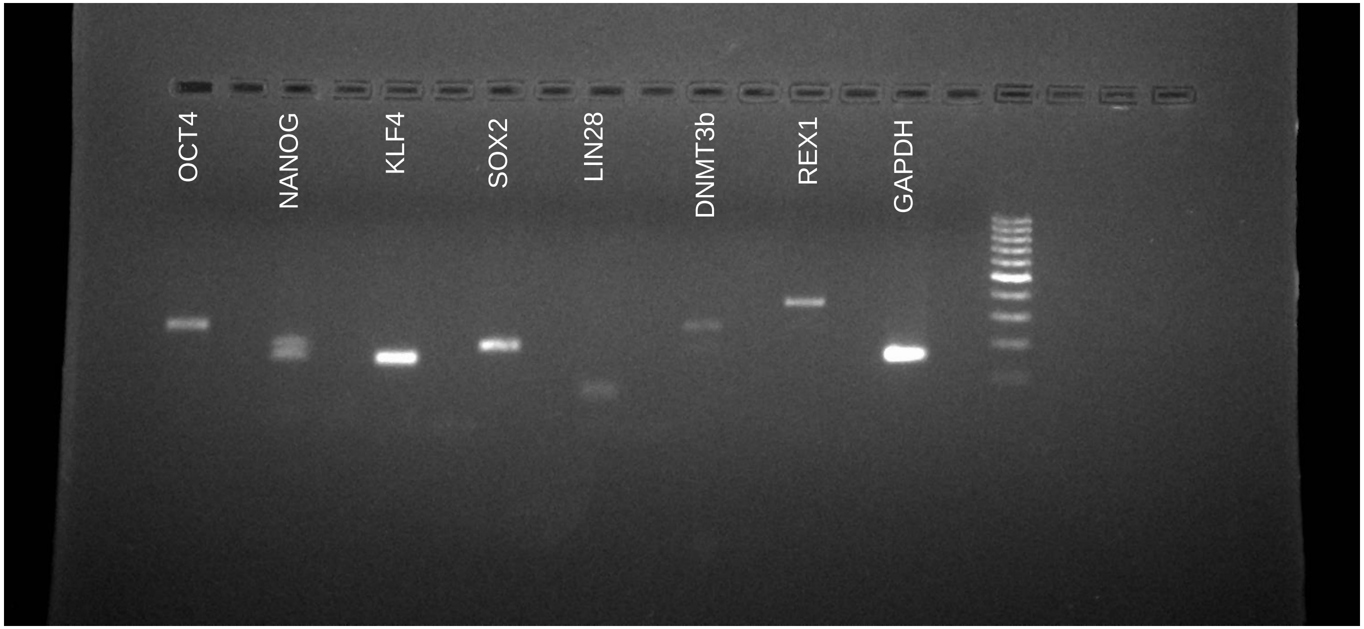
OCT4
NANOG
KLF4
SOX2
LIN28
DNMT3b
REX1
GAPDH

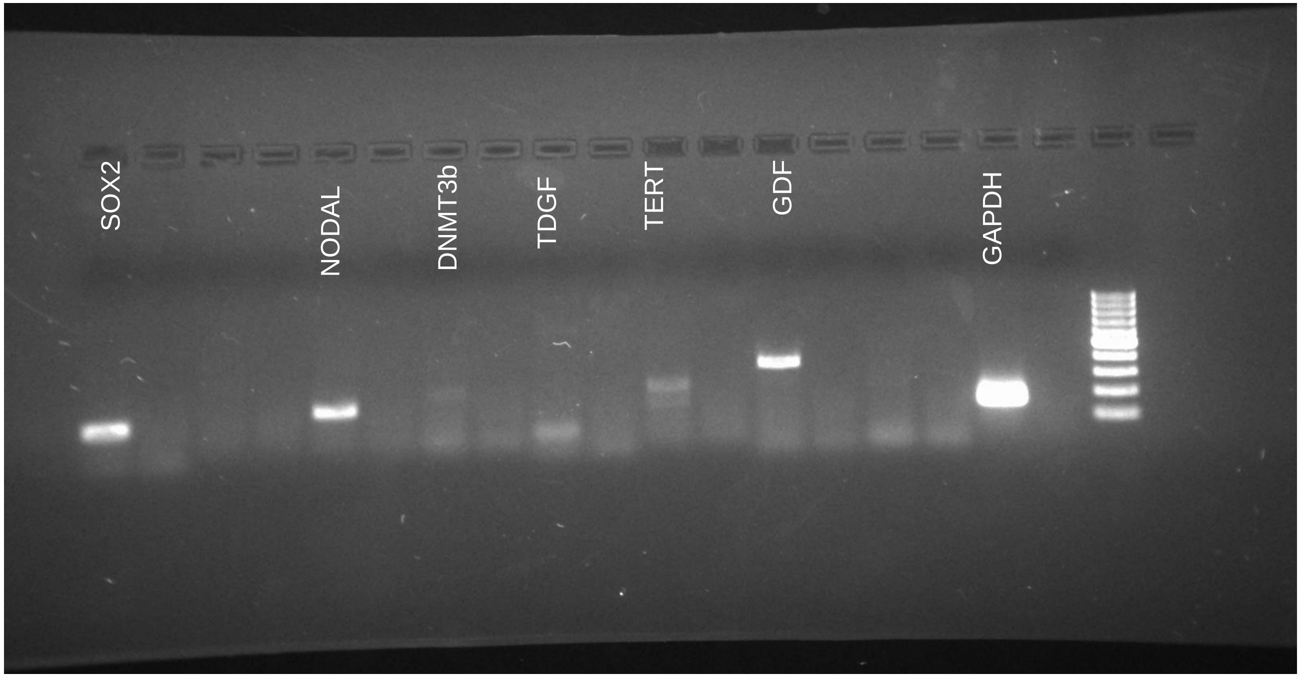
SOX2
NODAL
DNMT3b
TDGF
TERT
GDF
GAPDH

LQTS2-iPSC2

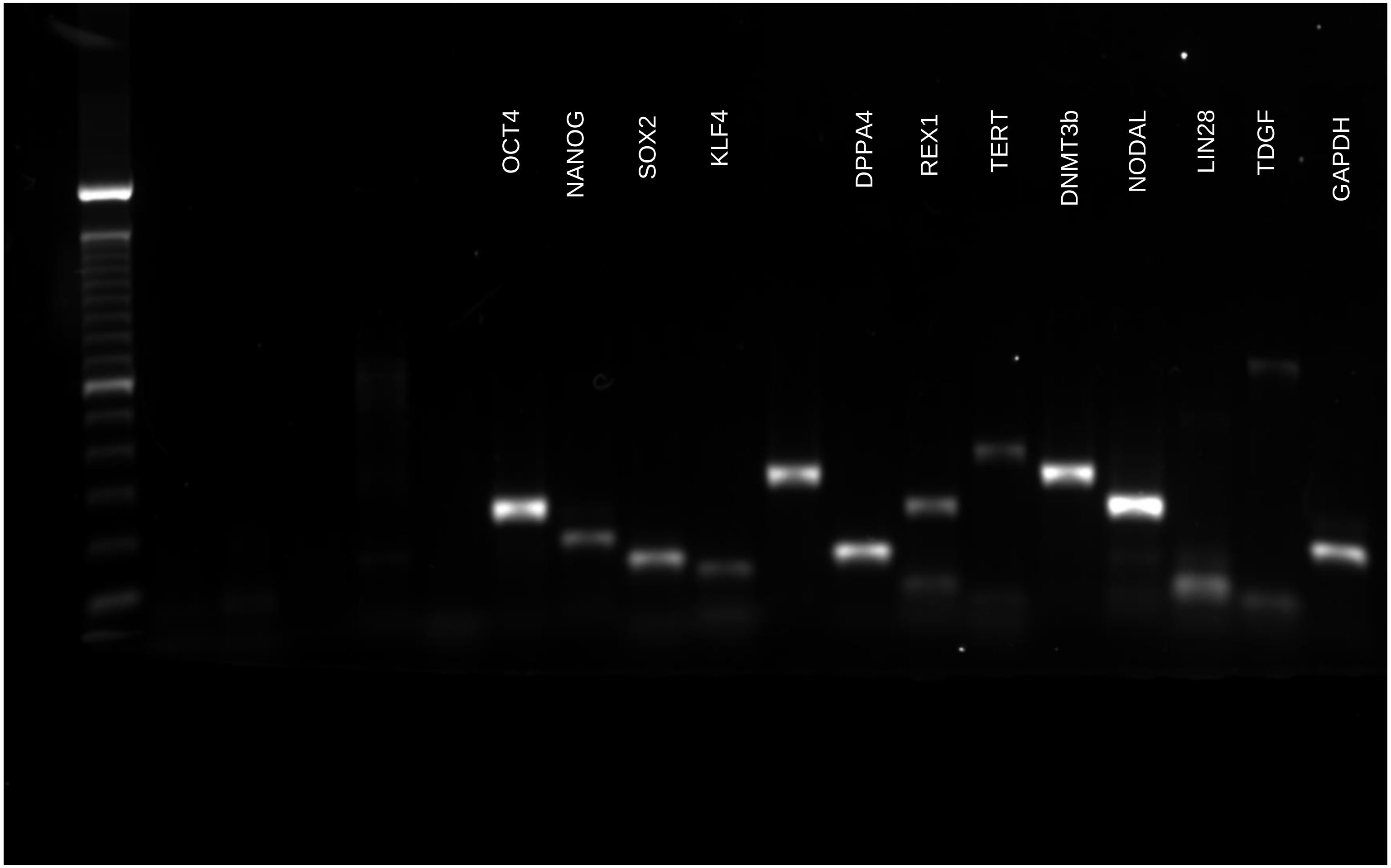
OCT4
NANOG
SOX2
KLF4
DPPA4
REX1
TERT
DNMT3b
NODAL
LIN28
TDGF
GAPDH

LQTS2-CRISPR

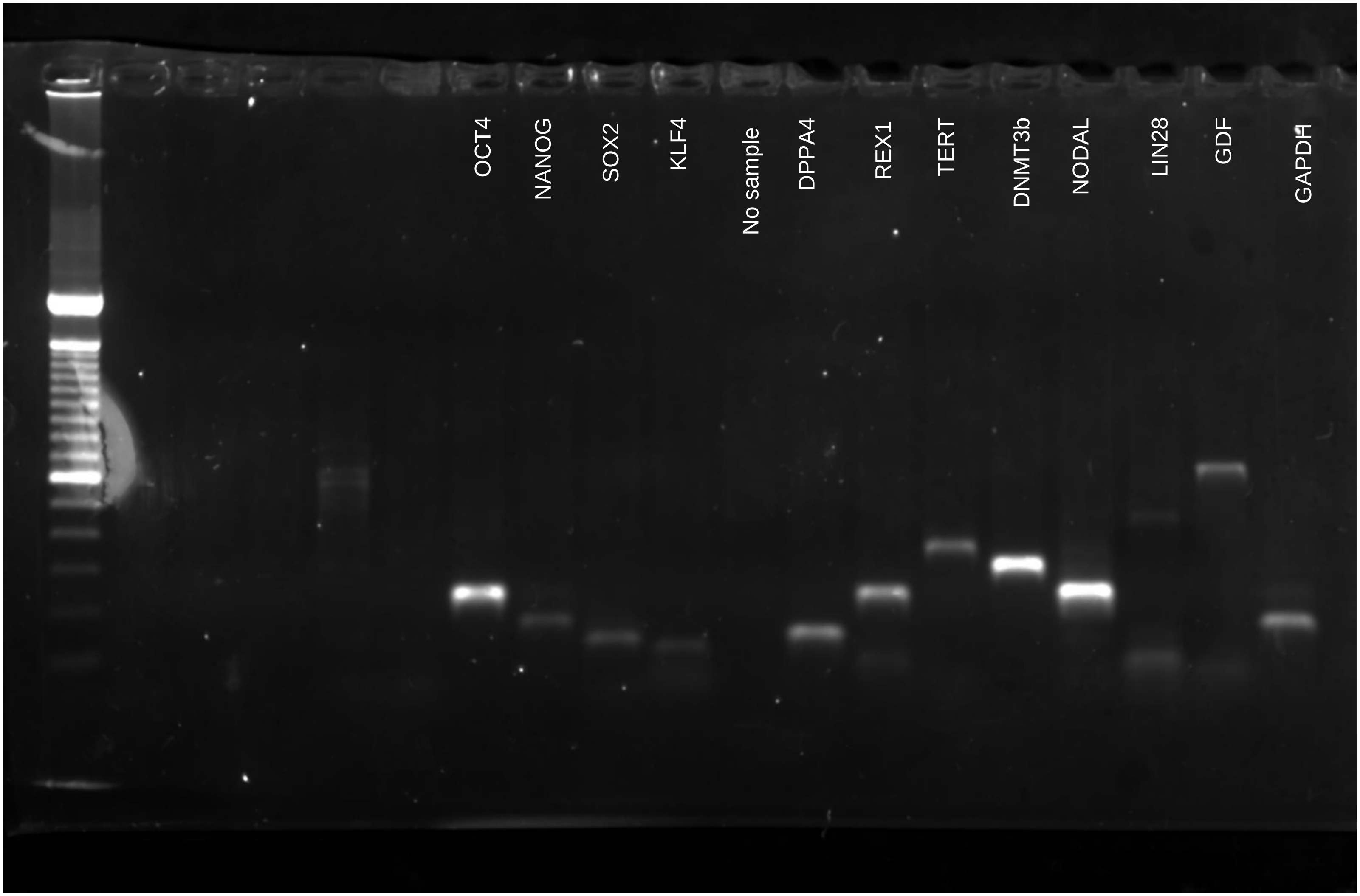

Supplement: Supplementary file 2 — Full length PCR gels [file 41598_2019_55837_MOESM2_ESM.pdf]
